# Quality Assessment and Physicochemical Characteristics of Bran Enriched *Chapattis*

**DOI:** 10.1155/2014/689729

**Published:** 2014-01-02

**Authors:** B. N. Dar, Savita Sharma, Baljit Singh, Gurkirat Kaur

**Affiliations:** ^1^Department of Food Technology, IUST, Awantipora, Jammu and Kashmir 191121, India; ^2^Department of Food Science and Technology, Punjab Agricultural University, Ludhiana 141004, India

## Abstract

Cereal brans singly and in combination were blended at varying levels (5 and 10%) for development of *Chapattis*. Cereal bran enriched *Chapattis* were assessed for quality and physicochemical characteristics. On the basis of quality assessment, 10% enrichment level for *Chapatti* was the best. Moisture content, water activity, and free fatty acids remained stable during the study period. Quality assessment and physicochemical characteristics of bran enriched *Chapattis* carried out revealed that dough handling and puffing of bran enriched *Chapattis* prepared by 5 and 10% level of bran supplementation did not vary significantly. All types of bran enriched *Chapattis* except rice bran enriched *Chapattis* showed nonsticky behavior during dough handling. Bran enriched *Chapattis* exhibited full puffing character during preparation. The sensory attributes showed that both 5 and 10% bran supplemented *Chapattis* were acceptable.

## 1. Introduction

*Chapattis* are a form of unleavened flatbreads of Indian/Eastern origin. They form an integral part of Indian diet, especially for those who have type 2 diabetes for whom white rice is considered less desirable because of its high GI. *Chapattis* and other flatbreads are popular in Europe also where they form a part of daily diet among members of ethnic minority groups who follow traditional dietary patterns. *Chapattis* are made from whole-wheat flour and cooked on hot flat open griddles. They can also be prepared by substituting wheat flour with other cereal or legume flours at different levels. This could result in either improvement or deterioration of texture and taste of *Chapattis*. Incorporation of cereal brans at proportions up to 10% has resulted in good quality *Chapattis*. The color and appearance of *Chapattis* were found to be good with substitution of wheat flour with up to 10% cereal brans [1]. Generally, *Chapatti* is prepared from whole-wheat flour obtained by grinding wheat in a disk mill (locally known as chakki). *Chapatti* quality can be assessed from its softness and flexibility which may be affected by flour protein quantity and quality. The *Chapatti* quality is also influenced by the dough consistency, which in turn depends mainly on the quantity of water added. *Chapatti* of good quality can be made by adding cereal brans even upto 10% [1]. The *Chapattis* made from composite flour showed higher extensibility even after 24-hour storage.

Bran is the hard outer layer of cereal grains, rich in a myriad of healthy phytochemicals, namely, phenolics, flavonoids, glucans, and pigments. Unfortunately, these nutrition-rich components are often discarded during milling out of ignorance, organoleptic reasons, and rancidity problems. Knowing the phytochemical constituents and pharmacological profile of bran is expected to give insight to their potential application in promotion of health. Cereal brans, the by-products obtained in large amounts in grain milling industry, considered as inedible material for humans, is mostly used as animal feed. However, brans are concentrated source of dietary fibre and other nutrients (proteins, B-vitamins, and minerals). Brans are generally composed mainly of insoluble cellulose and hemicellulose, with only about 5 percent soluble fibre, and have little hypercholesterolemia effect [2]. Bran contributes a pleasing, sweet, nutty flavour when added as a flavour enhancer in a variety of food products.

## 2. Materials and Methods

### 2.1. Raw Materials Used in the Investigation

Commercial wheat flour and oat bran (Baggry's India Ltd., New Delhi, India) were purchased from local market. Wheat bran was collected from Ludhiana Flour Mill, Ludhiana, India. Rice bran was purchased from “Ricela Health Foods Ltd.,” Dhuri, Punjab, India.

### 2.2. Product Preparation

*Chappati* was prepared by the addition of cereal brans (wheat, oat, and rice) singly and in combination (w : r : o:  :2 : 1.5 : 1.5) to wheat flour at 5 and 10% bran supplementation.

*Chapatti.* Cereal brans singly and in combination at 5 and 10% level were added to wheat flour and required quantity of water which were mixed manually to obtain dough of suitable consistency. The dough was rounded manually and kept for half an hour at room temperature. The dough was divided into four equal parts and moulded into circular *Chapattis *of 15.0 cm in diameter with rolling pin and board [3].

Traditional home baking procedure was followed to bake *Chapattis* on iron plate (Tawa). *Chapattis* were cooled and comparative evaluation was done using the following criteria which also included observations on dough handling properties. 

**Table:** 

*Characteristic*		*Score grade*
Dough handling		Nonsticky
		Sticky
		Slightly sticky
		Very sticky
		
Puffing of *Chapatti *		Full
		Partial
		Nil.

### 2.3. Physicochemical Composition of Prepared Products

Physicochemical composition of prepared product was determined by standard methods [4].

### 2.4. Texture and Colour Analysis

#### 2.4.1. Colour Analysis

**Figure 1:**
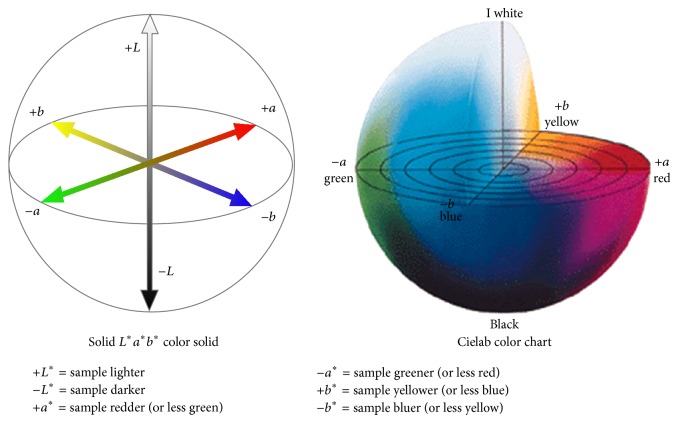


Colour analysis of cereal bran enriched *Chapatti* was done by using Hunter Lab colorimeter. The instrument was calibrated with the user supplied black plate calibration standard that was used for zero setting. Minolta supplied white calibration plates were used for white calibration setting. The sample was uniformly packed in clean petri plates with lid. The instruments were placed on the plate and three exposures at different places were conducted. Readings were displayed as *a*∗, *b*∗, and *L*∗ color parameters according to the CIELAB system of color measurement. The *a* value ranges from −100 (redness) to +100 (greenness) and the *b* value ranges from −100 (blueness) to +100 (yellowness), while the *L* value, indicating the measure of lightness, ranges from 0 (black) to 100 (white). The following guidelines indicate colour status (Figure 1).

#### 2.4.2. Texture Profile Analysis

Cutting force of *Chapatti* was evaluated by using texture analyser (Stable Micro Systems, Model TA-HDi, UK). Strips measuring 4 cm × 2 cm were cut from each *Chapatti*. One strip at a time was placed on the centre of the sample holder and the blade was allowed to cut the *Chapatti* strip. The force (N) required to cut *Chapatti* strip into two pieces was recorded. The speed was maintained at 1.70 mm/s.

### 2.5. Sensory Evaluation

Bran enriched products such as extruded snacks, breakfast cereal-Porridge, and *Chapatti* were evaluated for sensory attributes (appearance, colour, texture, flavor, and overall acceptability) through a panel of semi-trained judges using 9-point hedonic scale [5].

### 2.6. Water Activity

Water activity of bran enriched products was estimated using water activity meter having HygroLab 3 bench-top indicator (Rotrogenic Company).

### 2.7. Free Fatty Acids

Standard AOAC procedure [6] was followed for free fatty acids determination in cereal bran enriched products. Product sample (5 g) was taken in flask and 50 mL benzene was added and kept for 30 min for extraction of free fatty acids. After extraction, 5 mL extract, 5 mL benzene, 10 mL alcohol, and phenolphthalein as indicator were taken in flask and titrated against 0.02 N KOH till light pink colour disappeared:

(1) %FFA  (%  oleic  acid) =282×0.02 N  KOH×mL  of  alkali  used×dilution  factor1000×wt  of  sample  taken  ×100,

 
*moisture content* by method of AACC 2000 [4],
 
*total plate count* by method of Maturin and Peeler [7].

### 2.8. Statistical Analysis

Data collected from the aforesaid experiments was subjected to statistical analysis for standard error and Duncan's multiple range test using Minitab software. Values are mean of triplicates.

## 3. Results and Discussion

### 3.1. Bran Enriched *Chapatti*

#### 3.1.1. Quality Assessment

The quality evaluation of *Chapattis* prepared by different bran enriched levels is mentioned in Table 1. The pooled scores obtained by the various bran enriched levels of *Chapattis* for appearance, color, texture, and flavor were 7.92, 7.18, 7.68, and 8.10 for wheat bran, rice bran, oat bran, and bran in combination enriched *Chapattis*, respectively. All bran enriched *Chapattis* were in highly acceptable range. The overall acceptability at 5 and 10 percent level of supplementation was 7.65 and 7.80, respectively. However, it was 7.89 for control *Chapattis*. Butt et al. [1] reported that *Chapattis*, prepared by the addition of 10% bran, showed better performance and were quite comparable with whole-wheat flour regarding the proximate components and sensory attributes.

Dough handling characteristics of bran enriched *Chapattis* do not show much variation with respect to type of bran used. Except for rice bran incorporated dough for *Chapatti *(slightly sticky), all others showed nonsticky behavior during dough development. The puffing character also did not vary much. All types of bran enriched *Chapattis* showed full puffing except 10% rice bran enriched *Chapattis* in which partial puffing during *Chapatti* preparation was visualised.

### 3.2. Colour and Texture Analysis

The data presented in Table 2 depicted color and texture analysis of bran enriched *Chapatti*. Statistically significant (*P* ≤ 0.05) difference was observed in *L*∗ value of bran enriched *Chapatti*. *L*∗ value of various cereal bran enriched *Chapattis* was 64.37, 59.12, 60.04, and 61.92 for wheat, rice, oat, and bran in combination, respectively. *L*∗ value showed decreasing trend with increase in level of supplementation of cereal brans in *Chapattis*. The *L*∗ value of 66.83, 61.59, and 61.14 was observed at 0, 5, and 10% level of supplementation, which means slightly lower brightness at higher levels of supplementation. Reverse trend to that of *L*∗ value was observed in *a*∗ values of *Chapattis*. *Chapattis* having maximum *L*∗ values recorded minimum *a*∗ value and vice versa. *a*∗ value of wheat, rice, oat, and bran in combination was 4.18, 5.24, 4.71, and 4.28, respectively. With increase in level of supplementation, *a*∗ value (redness) increased from 3.14 at 0% level of supplementation to 5.11 at 10% level. The hue angle of bran enriched *Chapattis *varied from 72.36 to 75.56. Altan et al. [8] stated that, among the color parameters, the *L*∗ and *a*∗ values showed marked changes due to addition of tomato pomace. An increase in tomato pomace level decreased the *L* value of the sample and increased the *a*∗ value of samples. Also, increasing bran level supplementation resulted in a decrease in the *b*∗ value of *Chapattis*. A negative correlation was found between *a*∗ value and *b*∗ value of the enriched *Chapattis*.

Cutting force (N) reflects the texture of the *Chapattis* and it stimulates the biting action of the human teeth on *Chapattis* [9]. Cutting force (N) of various bran enriched *Chapattis* varied as 6.56 N, 5.92 N, 5.30 N, and 5.99 N for wheat, rice, oat, and bran in combination. Cutting force increased due to presence of more fibres at higher enrichment levels. At 0, 5, and 10 percent level of supplementation, the corresponding cutting force (N) was 5.25, 5.91, and 5.97 N, respectively. Manu and Prasada Rao [10] reported that cutting force of *Chapattis* prepared from different wheat varieties ranged from 4.22 to 6.96 N. Hemalatha et al. [11] also reported that the cutting force (N) for *Chapattis* made from different wheat varieties ranged between 4.22 and 6.67. The variation in cutting force might be because of variation in protein and fibre content of brans which determine the resistance offered by the samples.

### 3.3. Physicochemical Characteristics

*Physicochemical *characteristics of bran enriched *Chapatti* are elucidated in Table 3. Moisture content of bran enriched *Chapattis* ranged between 31.40 and 33.04%. A slight change in moisture content of *Chapattis* was observed with addition of bran. Increase in fibre content might have increased the water holding capacity of *Chapattis* and hence increased moisture content (%) with addition of bran. Yadav et al. [12] reported that moisture content (%) of bran enriched *Chapattis* was 31.0 percent while control had moisture content of 30.2 percent.

Water activity of bran enriched *Chapattis* ranged from 0.406 to 0.462. Maximum water activity was observed in rice bran enriched *Chapattis* (0.462) which was statistically at par with water activity of bran in combination enriched *Chapattis* (0.455). Water activity of oat and wheat bran enriched *Chapattis* was 0.429 and 0.406, respectively. It was observed from data that water activity of samples was positively correlated with moisture content and followed the same pattern. Increase in bran supplementation resulted in increase in water activity of bran enriched *Chapattis*. Manthey et al. [13] reported that water activity of bran/fibre enriched pasta increased with bran supplementation over control. The increase in water activity is correlated with increase in moisture content at higher levels of bran supplementation.

The data pertaining to free fatty acids (%) is presented in Table 3. The free fatty acids (%) of bran enriched *Chapattis* ranged from 0.057 to 0.085. The highest free fatty acids were recorded in rice bran enriched *Chapattis* (0.085%), being the lowest in wheat bran enriched *Chapattis* (0.057). It is also evident from the table that, with increase in level of bran supplementation, free fatty acids increased significantly. The free fatty acids (%) at 5 and 10 percent level of supplementation were 0.067 and 0.079 percent, respectively. Khan et al. [14] reported similar results regarding free fatty acids while studying development and evaluation of long shelf life ambient stable *Chapattis*.

Total plate count of bran enriched *Chapatti* is tabulated in Table 3. A significant variation (*P* ≤ 0.05) was observed in total plate count of bran extruded *Chapattis*. Total plate count of enriched *Chapattis* varied from 6.5 to 17 × 10^3^ cfu/g. It is also evident from the table that with increase in bran supplementation level, a slight increase in total plate content was observed. The mean value of total plate content for 5 and 10% level of supplementation was 10.25 × 10^3^ and 12.5 × 10^3^ cfu/g, respectively. The total plate count of *Chapattis* was under safe limits. Frazier and Westhoff [15] reported that total plate content increased from 2.3 × 10^2^ to 3.4 × 10^2^ cfu/g for flour.

## 4. Conclusions

Quality characteristics for *Chapatti *revealed that dough handling and puffing of bran enriched *Chapattis* prepared by 5 and 10% level of bran supplementation did not vary significantly. All types of bran enriched *chapattis* except rice bran enriched *chapattis* showed nonsticky behavior during dough handling. Bran enriched *chapatti*s exhibited full puffing character during preparation. The sensory attributes showed that both 5 and 10% bran supplemented *Chapattis* were acceptable. Physicochemicalcharacteristics of bran enriched *Chapattis* differed significantly (*P* ≤ 0.05). Rice bran enriched *Chapatti *recorded maximum moisture (%), water activity, and free fatty acids (%). With increase in level of supplementation, moisture, water activity, and free fatty acids increased. The future emphasis can be given on development of functional flatbreads which has got increased demand due to increase in health conscious consumer base.

## Figures and Tables

**Table 1a:** (a)

Type of bran & supplementation (%)	Dough handling	Puffing	Appearance	Color	Texture	Flavor	Overall acceptability
Control	NS	FP	8.2	7.6	8.06	7.7	7.89
Wheat bran							
5	NS	FP	8.1	7.9	7.76	7.6	7.84
10	NS	FP	8.2	8.1	7.7	8.0	8.00
Mean			**8.15**	**8.0**	**7.73**	**7.8**	**7.92**
Rice bran							
5	SS	FP	8.0	8.0	8.0	8.0	8.00
10	SS	PP	6.5	6.5	6.5	6.0	6.37
Mean			**7.25**	**7.25**	**7.25**	**7.0**	**7.18**
Oat bran							
5	NS	FP	7.25	7.25	7.0	7.5	7.25
10	NS	FP	8.0	8.0	8.25	8.25	8.12
Mean			**7.62**	**7.62**	**7.62**	**7.87**	**7.68**
In combination							
5	NS	FP	8.3	8.2	7.8	8.1	8.10
10	NS	FP	8.3	8.16	7.8	7.2	8.11
Mean			**8.30**	**8.18**	**7.8**	**7.65**	**8.10**

NS: nonsticky; S: slightly sticky; FP: full puffing; P: partially puffing.

**Table 1b:** (b)

Mean ± SE	Appearance	Color	Texture	Flavor	Overall acceptability
Bran					
Wheat	8.15^c^ ± 0.04	8.0^c^ ± 0.06	7.73^c^ ± 0.03	7.80^c^ ± 0.10	7.92^c^ ± 0.04
Rice	7.25^a^ ± 0.34	7.25^a^ ± 0.34	7.25^a^ ± 0.34	7.00^a^ ± 0.045	7.18^a^ ± 0.36
Oat	7.62^b^ ± 0.17	7.62^b^ ± 0.17	7.62^b^ ± 0.28	7.87^c^ ± 0.17	7.68^b^ ± 0.19
In combination	8.30^d^ ± 0.01	8.18^d^ ± 0.02	7.80^c^ ± 0.05	7.65^b^ ± 0.20	8.10^d^ ± 0.03
Level mean					
5%	7.91^b^ ± 0.12	7.83^b^ ± 0.11	7.64^b^ ± 0.08	7.80^b^ ± 0.08	7.65^a^ ± 0.22
10%	7.75^a^ ± 0.22	7.69^a^ ± 0.21	7.56^a^ ± 0.026	7.36^a^ ± 0.26	7.80^b^ ± 0.10

**Table 2a:** (a)

Type of bran & supplementation level (%)	*L* ^*^	*a* ^*^	*b* ^*^	Hue angle (°)	Cutting force (N)
Control	66.83	3.14	15.20	74.76	5.25
Wheat bran					
5	65.15	3.76	15.36	74.86	6.52
10	63.59	4.62	15.58	73.86	6.60
Mean	**64.37**	**4.18**	**15.47**	**74.36**	**6.56**
Rice bran					
5	61.32	4.08	17.15	76.31	5.88
10	56.92	6.41	16.52	73.48	5.96
Mean	**59.12**	**5.24**	**16.83**	**74.90**	**5.92**
Oat bran					
5	57.48	4.64	16.33	69.26	5.28
10	62.60	4.78	16.92	75.47	5.32
Mean	**60.04**	**4.71**	**16.62**	**72.36**	**5.30**
In combination					
5	62.41	4.04	16.93	76.10	5.96
10	61.44	4.63	15.74	75.01	6.02
Mean	**61.92**	**4.28**	**16.33**	**75.56**	**5.99**

**Table 2b:** (b)

Mean ± SE	*L* ^*^	*a* ^*^	*b* ^*^	Hue angle (°)	Cutting force (N)
Bran					
Wheat	64.37^c^ ± 0.35	4.18^a^ ± 0.20	15.47^a^ ± 0.05	74.36^b^ ± 0.23	6.56^d^ ± 0.02
Rice	59.12^a^ ± 0.98	5.24^b^ ± 0.52	16.83^d^ ± 0.14	74.90^c^ ± 0.63	5.92^b^ ± 0.02
Oat	60.04^b^ ± 1.14	4.71^c^ ± 0.03	16.62^c^ ± 0.13	72.36^a^ ± 1.39	5.30^a^ ± 0.01
In combination	61.92^d^ ± 0.22	4.28^d^ ± 0.11	16.33^b^ ± 0.27	75.56^d^ ± 0.25	5.99^c^ ± 0.02
Level mean					
5%	61.59^b^ ± 0.83	4.13^b^ ± 0.31	16.44^b^ ± 0.21	74.14^a^ ± 0.86	5.91^a^ ± 0.13
10%	61.14^a^ ± 0.77	5.11^a^ ± 0.08	16.19^a^ ± 0.17	74.46^b^ ± 0.24	5.97^b^ ± 0.14

**Table 3a:** (a)

Type of bran & supplementation level (%)	Moisture (%)	Water activity (*a*_*w*_)	Free fatty acids (%)	TPC (×10^3^ cfu)
Control	30.31	0.456	0.040	18
Wheat bran				
5	32.33	0.442	0.052	12
10	30.47	0.483	0.063	14
Mean	**31.40**	**0.462**	**0.057**	**13**
Rice bran				
5	32.50	0.428	0.074	15
10	33.58	0.430	0.097	19
Mean	**33.04**	**0.429**	**0.085**	**17**
Oat bran				
5	32.15	0.391	0.076	8
10	33.23	0.422	0.084	10
Mean	**32.69**	**0.406**	**0.085**	**9**
In combination				
5	32.65	0.455	0.074	6
10	32.38	0.456	0.074	7
Mean	**32.52**	**0.455**	**0.074**	**6.5**

**Table 3b:** (b)

Mean ± SE	Moisture	Water activity	Free fatty acids	TPC (×10^3^ cfu)
Bran				
Wheat	31.40^a^ ± 0.42	0.462^d^ ± 0.009	0.057^a^ ± 0.002	13^c^
Rice	33.04^d^ ± 0.24	0.429^b^ ± 0.001	0.085^d^ ± 0.005	17^d^
Oat	32.69^c^ ± 0.24	0.406^a^ ± 0.006	0.077^c^ ± 0.003	9^b^
In combination	32.52^b^ ± 0.06	0.455^c^ ± 0.001	0.074^b^ ± 0.001	6.5^a^
Level mean				
5%	32.68^d^ ± 0.10	0.429^a^ ± 0.007	0.067^a^ ± 0.003	10.25^a^
10%	32.14^a^ ± 0.33	0.448^b^ ± 0.007	0.079^b^ ± 0.004	12.50^b^
